# Selecting the Proper Transcatheter Aortic Valve Replacement Device in East Asians With a Small Aortic Annulus

**DOI:** 10.1016/j.jacasi.2024.11.002

**Published:** 2025-01-14

**Authors:** Ying-Hwa Chen, Howard C. Herrmann

**Affiliations:** a Cardiovascular Center, Taipei Veterans General Hospital, Taipei, Taiwan; b School of Medicine, National Yang-Ming Chiao-Tung University, Taipei, Taiwan; c Cardiovascular Division, Perelman School of Medicine at the University of Pennsylvania, Philadelphia, Pennsylvania, USA

**Keywords:** aortic stenosis, prosthesis-patient mismatch, small aortic annulus

East Asian populations undergoing transcatheter aortic valve replacement (TAVR) exhibit unique anatomical and procedural characteristics compared with non-Asian populations. Specifically, Asian patients tend to have lower body mass index, smaller body surface area (BSA), and smaller aortic valve annular size, necessitating the use of smaller TAVR prostheses. The East Asian region has a population of more than 2 billion people, representing almost 30% of the world’s residents. However, there is a notable under-representation of this region in clinical studies.

There is significant heterogeneity within the East Asian demographic. The median BSA in Chinese,^1^ Taiwanese,^2^ and Koreans^3^ is 1.6 m^2^, between Japanese (1.4 m^2^)^4^ and Caucasians (1.8-1.9 m^2^). Computed tomography-derived annulus diameters were reported as 23.1 mm in Taiwanese and Koreans^2^^,^^3^ and 22.0 to 22.4 mm in Japanese individuals.^4^^,^^5^ In contrast, major trials and registries in Western populations report a range of 24.2 to 24.6 mm. Small TAVR prostheses (20-/23-mm Sapien XT/Sapien 3 or 23-/26-mm CoreValve/Evolut) were utilized in 37% to 70% of East Asian populations^2^, ^3^, ^4^, ^5^, ^6^ compared with 21% to 32% in Western populations.

## Small Aortic Annuli

Patients with small aortic annuli (SAA) are at higher risk for residual gradients and prosthesis-patient mismatch (PPM), which are linked to adverse cardiovascular events, including death and heart failure. SAA is typically defined as an annular diameter ≤23 mm, an area ≤400 to 415 mm^2^, or a perimeter ≤72 mm on computed tomography imaging.

## Incidence and Predictors of PPM

**Figure 1:**
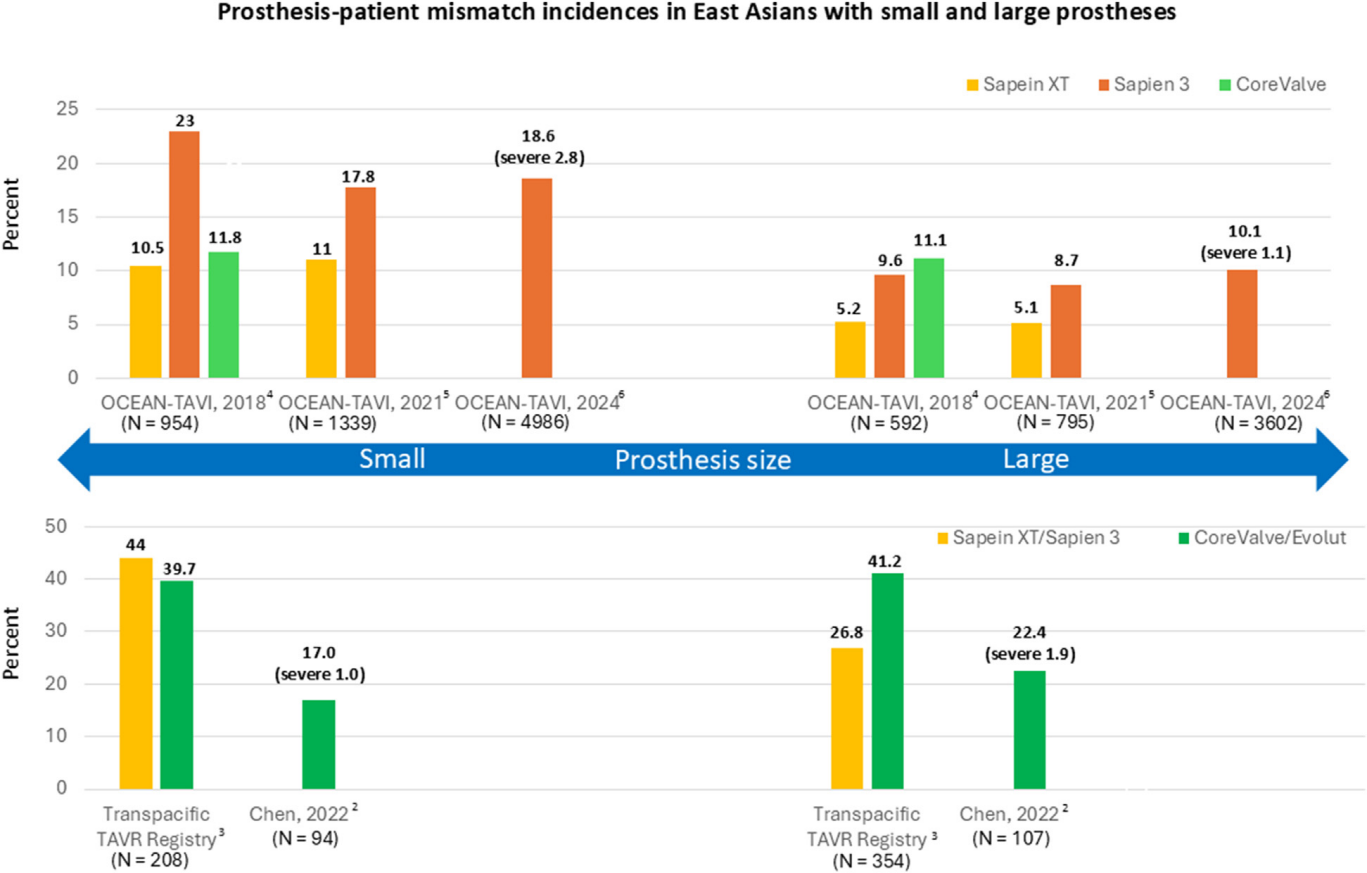
Prosthesis-Patient Mismatch Incidence in East Asian Patients With Different Prostheses The incidence of prosthesis-patient mismatch in East Asian patients treated with either small (20-/23-mm Sapien XT/Sapien 3 or 23-/26-mm CoreValve/Evolut) or large prostheses (26-/29-mm Sapien XT/Sapien 3 or 29-/31-/34-mm CoreValve/Evolut platform).

Although East Asians have smaller aortic valve annuli, their considerably lower BSA compared with White individuals results in a lower incidence of PPM. Figure 1 summarizes the incidences of PPM in East Asian patients treated with small or large prostheses (26-/29-mm Sapien XT/Sapien 3 or 29-/31-/34-mm CoreValve/Evolut platform).

Insights into PPM after TAVR in Japan were drawn from 3 studies using the OCEAN-TAVI (Optimized transCathEter vAlvular iNtervention) registry across various time periods.^4^, ^5^, ^6^ The average BSA of 1.4 m^2^ among Japanese patients highlighted its role in reducing the incidence of PPM within this population. The initial study included 1,546 patients, 71% women, with 90% receiving balloon-expandable valves (BEVs), mainly Sapien XT.^5^ Small prostheses were used in 61.7% of patients with either 20/23-mm Sapien XT/Sapien 3 or 26-mm CoreValve. Moderate and severe PPM were observed in 8.9% and 0.7% of patients, respectively, with Sapien 3 independently linked to PPM (OR: 2.73; *P =* 0.0002). In the subsequent registry of 2,134 patients treated with BEV, 69% were women, and 62.7% received smaller prostheses.^4^ Moderate and severe PPM occurred in 10.0% and 1.0% of cases, respectively. Multivariate analysis identified Sapien 3 over Sapien XT (OR: 1.92; *P =* 0.0003) and the use of small prostheses as independent predictors of PPM. Possible factors of the higher incidence of PPM with the Sapien 3 include the additional material of the outer skirt, the lower area oversizing of the S3, and its hyperboloid shape. In a recent study of 8,750 patients treated with Sapien 3, 61% were women and 58% received small prostheses.^6^ Moderate and severe PPM were observed in 12.8% and 2.0% of patients, respectively. For patients treated with small prostheses, moderate and severe PPM occurred in 27.6% and 6.6% with 20-mm Sapien 3, and 14.6% and 2.4% with 23-mm Sapien 3, respectively. In those with larger prostheses, moderate and severe PPM were observed in 9.9% and 1.1% with 26-mm Sapien 3, and 5.2% and 0.9% with 29-mm Sapien 3, respectively.

The Multiracial Transpacific TAVR Registry included 562 Asian patients (mainly Korean), 49% women, with an average BSA of 1.6 m^2^. BEVs, primarily Sapien 3, were used in 83% of cases, with 37% receiving small prostheses.^3^ The incidence of PPM was significantly lower in Asians (33.6%; moderate 26.5%; severe 7.1%) compared with non-Asians (54.5%; moderate 29.8%; severe 24.7%). In a Korean TAVR registry of 660 patients treated either with BEV or self-expanding valve (SEV), 10.6% had a small annulus diameter of ≤20 mm.^7^ Those with a small annulus diameter had significantly higher rate of PPM (33.3%) than those with larger annuli (19.2%) at 30 days, although this difference was not significant at 1 year. In a prospective single-center study of 201 Taiwanese TAVR patients treated exclusively with SEV, 56% were women, with an average BSA of 1.6 m^2^.^2^ Small prostheses were used in 47%. Moderate and severe PPM were observed in 18.4% and 1.5% of patients, respectively, with no significant link to valve size.

The limitation of registries is that patient enrollment and data entry are voluntary. Effective orifice area (EOA) measurements were largely site-measured without standardized methods.^3^, ^4^, ^5^, ^6^, ^7^ Only 1 study^2^ utilized an independent core laboratory for post-TAVR echocardiogram analysis.

## Impact of PPM and Hemodynamic Performance

In the OCEAN-TAVI Japanese registry of 1,546 patients, 1-year all-cause and cardiovascular mortality were similar between the PPM and non-PPM groups (10.2% vs 8.3% and 3.9% vs 2.3%, respectively), with no significant difference based on PPM severity.^5^ Another study of 2,134 Japanese patients also found no difference in cumulative all-cause and cardiovascular mortality at a median follow-up of 721 days.^4^ The Multiracial Transpacific TAVR Registry reported similar 1-year rates of death, stroke, or rehospitalization between PPM and non-PPM groups (27.5% vs 28.1%), consistent across both Asian and non-Asian patients.^3^ However, a Taiwanese study of 201 patients with PPM showed higher risks of all-cause death (adjusted HR: 1.95; *P =* 0.027), cardiovascular mortality (adjusted HR: 3.38; *P =* 0.043), and rehospitalization for heart failure (adjusted HR: 2.40; *P =* 0.025) at midterm (median 30.4 months) follow-up.^2^

The heterogeneity of the study results may be related to the difference in patient demographics and valve types. Registry limitations include procedural complications and clinical outcomes reported by individual physicians without an events committee, causing selection bias. Despite propensity matching, uncaptured clinical factors are concerning. Additionally, small sample sizes and short follow-ups limit the study's power to detect differences in clinical endpoints between patients with and without PPM. These studies utilized measured PPM that may include the effects of low flow, which is more prevalent early after TAVR and results in an overestimation of true PPM. To overcome this limitation, predicted PPM has been proposed. However, this measure also has limitations, such as relying on echocardiographic measurements and not accounting for inadequate expansion, asymmetric prosthesis shape, leaflet material, or frame recoil.

The prevalence of postprocedural mean pressure gradient (mPG) ≥20 mm Hg varied over time in the OCEAN-TAVI Japanese registry.^4^, ^5^, ^6^ The initial study, primarily utilizing the Sapien XT valve, reported this in 1.9% of patients, with a significantly higher rate observed in PPM patients compared with non-PPM patients (8.1% vs 1.3%; *P <* 0.0001).^5^ The second registry found a prevalence of 2.4% with the Sapien XT and 6.5% with the Sapien 3, observed only in patients receiving smaller prostheses, with PPM patients being more affected (15.6% vs 2.6%; *P <* 0.0001).^4^ A more recent registry involving the Sapien 3 valve showed a prevalence of 26.3% in the 20-mm prosthesis, 7.1% in the 23-mm, 2.2% in the 26-mm, and 0.9% in the 29-mm prosthesis.^6^ The Multiracial Transpacific TAVR Registry, which predominantly used the Sapien 3, reported a prevalence of 12.1% in Asians (primarily Koreans) and 8.1% in non-Asians, with a significantly higher rate observed in PPM patients compared with non-PPM patients (22.8% vs 6.7%; *P <* 0.001) in Asia.^3^ A Taiwanese study using the SEV valve found a prevalence of 1.0%.^2^

### Comparison of SEV vs BEV in Patients With SAA

The Evolut supra-annular SEV has improved hemodynamics relative to BEV, because of the funnel shape (with supra-annular leaflets), less tissue within the frame, and other engineering features. In the randomized SMART trial comparing SEV to BEV in SAA patients undergoing TAVR, SEV showed a 0.5-cm^2^ larger EOA, 8-mm Hg lower mPG, and 0.19-higher Doppler velocity index compared with BEV at 1 year. This study was conducted in 87 North American, European, and Middle-Eastern countries, and these findings may not directly translatable to the East Asian population.

Table 1 summarizes mPG, indexed EOA, and PPM incidence in East Asian patients with SAA treated with SEV or BEV. In the OCEAN-TAVI Japanese registry of 974 patients, 59% had SAA (≤23 mm), with 487 receiving Sapien 3 and 89 receiving Evolut R; 87% were women.^8^ Propensity score matching included 69 patients in each group. The Evolut R group consistently showed lower mPG and higher indexed EOA at discharge and 1 year compared with the Sapien 3 group. Moreover, the Sapien 3 group exhibited a decrease in indexed EOA at 1 year. PPM incidence was numerically higher in the Sapien 3 group but not significantly different, and 1-year mortality rates were similar between groups. In a subset of 205 patients (21%) with extremely small annuli (annular diameter ≤21 mm), the Evolut R group again had better mPG and indexed EOA at discharge and 1 year. The incidence of moderate PPM was lower in the Evolut R group at 1 year, while severe PPM and mortality rates were similar between groups. In a Japanese single-center study of 302 patients, 16% had annular areas <330 mm^2^. Patients with annular areas <330 mm^2^ treated with Evolut R/PRO had significantly lower mPG, higher indexed EOA, and lower PPM incidence compared to those using Sapien 3 at 30 days and 1 year.^9^

**Table 1:** Comparison of Self-Expanding and Balloon-Expandable Valve Performance in East Asian Patients With Small Aortic Annuli

	Discharge or 30 Days				1 Year			
	Overall	SEV	BEV	*P* Value	Overall	SEV	BEV	*P* Value
OCEAN-TAVI Japanese Registry^8^								
Small annulus (annulus diameter ≤23 mm), n	138	69	69		97	47	50	
Mean PG, mm Hg	11.0	9.0	12.0	<0.001	10.0	9.0	12.0	<0.001
Indexed EOA, cm^2^/m^2^	1.12	1.20	1.08	0.01	1.04	1.21	0.96	<0.001
Moderate PPM	17 (12.9)	5 (7.7)	12 (17.9)	0.08	21 (21.6)	7 (14.9)	14 (28.0)	0.12
Severe PPM	3 (2.3)	1 (1.5)	2 (3.0)	1.00	2 (2.1)	0	2 (4.0)	0.50
Extreme small annulus (≤21 mm), n	205	45	160		145	29	116	
Mean PG, mm Hg	12.5	9.0	13.6	<0.001	13.1	8.0	15.0	<0.001
Indexed EOA, cm^2^/m^2^	1.07	1.17	1.04	0.002	1.00	1.20	0.97	<0.001
Moderate PPM	34 (17.0)	4 (9.1)	30 (19.2)	0.11	35 (24.1)	2 (6.9)	33 (28.4)	0.015
Severe PPM	4 (2.0)	1 (2.3)	3 (1.9)	1.00	5 (3.4)	0	5 (4.3)	0.26
Japanese single-center study (annular area <330 mm^2^),^9^ n	43	11	32		39	11	28	
Mean PG, mm Hg		9.3	15.0	<0.001		8.2	17.4	<0.001
Indexed EOA, cm^2^/m^2^		1.0	0.8	0.09		0.9	0.7	0.046
Moderate PPM		2 (18)	10 (31.3)	0.018		1 (9.1)	8 (28.6)	0.02
Severe PPM		0	9 (28)			0	10 (36)	
Korean single-center study (annulus diameter ≤23 mm),^10^ n	70	45	25		53	35	18	
Mean PG, mm Hg	9.9	8.9	11.7	0.005	10.6	8.5	14.7	<0.001
Indexed EOA, cm^2^/m^2^	1.10	1.18	0.95	0.001	1.01	1.12	0.81	<0.001
Moderate PPM	12 (17.1)	4 (8.9)	8 (32.0)	0.009	10 (18.9)	4 (11.4)	6 (33.3)	<0.001
Severe PPM	1 (1.4)	0	1 (4.0)		6 (11.3)	0	6 (33.3)	
Korean TAVR registry (annulus diameter ≤20 mm),^7^ n	57	34	23		47	31	16	
Mean PG, mm Hg		9.9	15.5	0.001		10.9	15.7	0.007
EOA, cm^2^/m^2^		1.04	0.90	0.029		1.06	0.97	0.240
Moderate PPM		6 (17.1)	9 (39.1)	0.163		5 (16.7)	3 (18.8)	0.462
Severe PPM		3 (8.6)	2 (8.3)			1 (3.3)	2 (12.5)	

Values are median, mean, or n (%).

BEV = balloon-expandable valve; EOA = effective orifice area; PG = pressure gradient; PPM = prosthesis-patient mismatch; SEV = self-expanding valve.

In a Korean single-center study of 70 patients with SAA (annular diameter ≤23 mm), 93% were women. The Evolut R/PRO group exhibited significantly lower mPG and higher indexed EOA at discharge and 1 year, with a lower incidence of PPM compared with the Sapien 3 group.^10^ In a Korean TAVR registry of 660 patients, those with an annulus diameter ≤20 mm treated with SEV showed lower mPG and higher EOA at 30 days, with mPG differences persisting at 1 year, while PPM incidence was similar between SEV and BEV groups at both time points.^7^

## Conclusions

Asian patients typically have smaller aortic annuli, necessitating the use of smaller prostheses during TAVR. Despite these smaller annuli, lower BSA in Asian populations contributes to a reduced incidence of PPM compared with Western populations. PPM, which occurs when the prosthetic valve is inadequately sized relative to the patient's body, poses significant risks, including suboptimal hemodynamic performance and increased long-term mortality.

In East Asian populations, 47% to 59%^2^^,^^9^ have an annular diameter ≤23 mm, leading to smaller TAVR prostheses in 37% to 70% of cases.^2^, ^3^, ^4^^,^^6^^,^^7^ Japanese patients, with an average BSA of 1.4 m^2^, display varying PPM rates based on valve type and size: Sapien XT (8.5%-8.8%), Sapien 3 (14.6%-18.0%), and CoreValve (11.5%).^4^^,^^6^^,^^7^ Notably, the use of Sapien 3 and 20-/23-mm BEV are significant predictors of PPM.^4^^,^^6^ In contrast, Korean patients, who have a larger average BSA of 1.6 m^2^, show a 33.6% PPM rate with Sapien 3,^3^ while Taiwanese patients utilizing SEV experience lower PPM rates.^2^

Overall, PPM incidence is significantly higher among patients receiving 20-/23-mm BEV, ^4^^,^^6^^,^^7^ while it remains consistent across various SEV sizes.^2^ The impact of PPM on mortality was inconsistent across studies.^2^, ^3^, ^4^^,^^6^ SEVs demonstrate superior hemodynamic performance in East Asian patients with small aortic annuli, underscoring the need for customized device selection and procedural planning to mitigate PPM risks in this population.

## Funding Support and Author Disclosures

Dr Chen has served as proctor and received consulting fees from Medtronic. Dr Herrmann has received institutional research funding from Abbott, Boston Scientific, Edwards Lifesciences, Highlife, Medtronic, and WL Gore; has received consulting fees from Edwards Lifesciences, Medtronic, Wells Fargo, and WL Gore; and has equity in Holistick Medical and Microinterventional Devices.
